# Tumour-agnostic drugs in paediatric cancers

**DOI:** 10.1038/s41416-020-0770-5

**Published:** 2020-03-12

**Authors:** Julia C. Chisholm, Fernando Carceller, Lynley V. Marshall

**Affiliations:** Paediatric and Adolescent Oncology Drug Development Team, Children and Young People’s Unit, Royal Marsden Hospital and Institute of Cancer Research, Sutton, UK

**Keywords:** Molecular medicine, Paediatric cancer

## Abstract

The recognition that new cancer drugs can be truly tumour-agnostic based on mechanism-of-action is important for paediatric cancers, where access to novel targeted therapies developed for adult indications has sometimes been problematic. The recently approved drug larotrectinib is an excellent case study of the development of a tumour-agnostic drug relevant to children.

## Main

There was much excitement within paediatric and adult oncology communities when on 19 September 2019 the European Medicines Agency (EMA) granted conditional marketing authorisation to the tropomyosin receptor kinase (TRK) inhibitor larotrectinib for use in adults and children with a documented neurotrophic tyrosine receptor kinase (NTRK) gene fusion whose disease has spread or cannot be surgically removed and who have no other satisfactory treatment options. The Food and Drug Administration (FDA) had granted accelerated approval in November 2018: the ruling from EMA extends availability to Europe. However, in England the excitement has been tempered by the decision released on 17 January 2020 by its health technology assessment (HTA) body, the National Institute for Health and Care Excellence (NICE), to approve the drug neither for routine National Health Service (NHS) use nor for the Cancer Drugs Fund (CDF) based on its cost-effectiveness. A publication from senior members of the NICE team and CDF outlining the challenges to HTA bodies of assessing clinical efficacy and value for money of tumour-agnostic drugs and the need for post-authorisation data collection paved the way for release of the NICE decision.^1^

The enthusiasm generated by the EMA decision was based on several factors: larotrectinib is the first drug to reach marketing authorisation following intentional development for a tumour-agnostic indication;^2^ the drug is well tolerated and its clinical activity in patients with NTRK gene fusions is histiotype independent and genuinely very promising;^3^ finally, and of great importance, paediatric development of larotrectinib intentionally commenced early during adult development so the evidence presented to the EMA included meaningful numbers of children.^2,3^

The concept of tumour-agnostic drugs, those that treat tumours according to the key molecular aberration(s) underlying the cancer rather than the histological tissue type involved, has been long recognised. With increasing understanding of the oncogenic drivers of cancer, it has become apparent that diverse tumour types with different cells of origin may share oncogenic drivers.

The first ever approved tumour-agnostic drug was the monoclonal anti-programmed cell death protein-1 antibody pembrolizumab.^2^ Although developed and initially approved in 2014 for specific indications including advanced melanoma, in 2017 the FDA broadened the approval of this immune checkpoint inhibitor to include adult and paediatric patients with microsatellite instability-high and mismatch repair-deficient tumours. In reality, these latter tumour-agnostic indications are rare in children, and other drugs in the same class (e.g. nivolumab, atezolizumab, durvalumab) are currently approved only for (different) tumour-specific indications.

Larotrectinib was the next drug to receive tumour-agnostic approval and entrectinib, an inhibitor of TRK, ROS1 and ALK, was FDA approved in 2019 for selected patients aged >12 years with NTRK fusions and adults with metastatic non-small-cell lung cancer whose tumours are ROS1 positive.^2^ Entrectinib was granted EMA Priority Medicines Designation in October 2017, potentially accelerating assessment of its European Marketing Authorisation Application and is currently being tested in paediatric patients with NTRK and ROS aberrations (NCT02650401). Continued FDA approval of both larotrectinib and entrectinib may be contingent on verification and description of clinical benefit in confirmatory trials.

NTRK gene fusions are found in about 1% of solid tumours across multiple different adult histiotypes. In children, NTRK fusions are characteristic of infantile fibrosarcoma and cellular congenital mesoblastic nephroma, occur very frequently (>75%) in secretory breast carcinoma and mammary analogue secretory carcinoma of the salivary gland, are frequent (10–40%) in infant high-grade gliomas, spitzoid melanoma and papillary thyroid tumours, and can occur rarely (<5%) in undifferentiated/spindle cell soft tissue sarcomas and inflammatory myofibroblastic tumours.^4^ The fusions result in constitutive activation of the TRK A/B/C protein kinases, protein products of the genes NTRK 1/2/3, respectively. The objective response rate to larotrectinib in 122 patients whose tumours harbour a NTRK fusion was 81% across all ages and independent of tumour histiotype.^3^ Thus the authorisation of larotrectinib realises a paradigm shift towards the availability of drugs developed and authorised based on their mechanism of action.^5^

The clinical development of larotrectinib is exemplary from a paediatric standpoint. The first clinical trial of the drug was the LOXO-TRK-14001 Phase 1 trial, which included 70 adult patients ≥18 years with metastatic solid tumours.^6^ All eight patients with a documented NTRK fusion showed an objective response. No patients without NTRK fusion/amplifications responded. The adult Phase 2 study (NAVIGATE; NCT02576431) in patients with documented NTRK fusions commenced within 18 months of the start of Phase 1 and included adolescents ≥12 years. The paediatric Phase 1/2 study (SCOUT study; NCT02637687) followed close on its heels, based on the responses seen in adults with NTRK fusions and, crucially, facilitated by the early development of a liquid oral formulation, since many of these NTRK fusion-positive tumours occur in very young children. The SCOUT study enrolled children and adolescents aged 1 month to 21 years with locally advanced or metastatic solid tumours, including central nervous system tumours, regardless of NTRK fusion status. The drug was well tolerated in children, the maximum tolerated dose was not reached, and the recommended phase II dose was 100 mg/m^2^ bd. No patients without NTRK fusions responded. In contrast, the objective response rate was 93% among 15 patients with NTRK fusions.^7^ The Phase 2 part is ongoing.

Targeted agents are usually developed alongside a companion diagnostic assay. This has not been the case for larotrectinib. Immunohistochemistry with TRK A, C or pan-TRK antibodies may indicate increased TRK expression suggesting the need for further elucidation.^4^ Clinical testing for the NTRK3-ETV6 translocation using fluorescent in situ hybridisation is routine in suspected infantile fibrosarcoma and cellular congenital mesoblastic nephroma. But it remains a potential challenge to identify NTRK fusions across a wide range of solid tumours where the incidence is low.^4^ In the UK, testing for NTRK fusions will be routine in the test directory for the planned NHS genomic medicine service for all newly diagnosed paediatric solid malignancies in children and young people and within the Stratified Medicine Paediatrics (ISRCTN21731605) molecular profiling programme in relapsed solid tumours^8^ that aims to facilitate matching of molecular aberrations in relapsed tumours with availability of targeted drugs within available clinical trials, such as the multi-arm, adaptively designed, proof-of-concept ESMART trial (NCT02813135). Early knowledge of the presence of NTRK fusions and other targets of tumour-agnostic drugs should benefit individual patients.

Other potential tumour-agnostic drugs relevant to children are currently being tested in the clinic.^2^ These include the pan-fibroblast growth factor receptor (FGFR) inhibitor TAS-120, the RET inhibitor LOXO 292 and second-generation TRK inhibitors. The poly (ADP-ribose) polymerase inhibitor olaparib, currently approved for ovarian, fallopian tube, peritoneal and breast BRCA1 and 2-related malignancies and checkpoint kinase inhibitors in tumours with somatic or germline TP53 mutations, are also in clinical trials in children, so we will soon gain further understanding of whether these drugs have true tumour-agnostic potential.

Some common potent oncogenic drivers, such as BRAF point mutations, do not always predict response to a relevant targeted agent across adult tumour types,^2^ but promising results in a variety of paediatric V600E mutated tumours (high- and low-grade glioma, papillary thyroid cancer, Langerhans cell histiocytosis, melanoma) raise the intriguing question of whether BRAF inhibitors may be tumour agnostic in children. By contrast, response of children to ALK inhibitors such as crizotinib and ceritinib seems to be influenced by both histiotype and specific oncogenic aberration in paediatric patients with ALK gene aberrations.^9^ For this reason, many important targeted agents are not truly tumour agnostic.

Historically, the development of drugs for paediatric cancers has lagged behind development for adult indications.^10^ The European Paediatric Regulation (2007) that requires pharmaceutical companies to develop a paediatric investigation plan or apply for a class or product-specific waiver has improved access by children to novel agents, but development of drugs for children based on mechanism of action has been slow to take off.^5^ The ACCELERATE international multi-stakeholder platform^10^ through its FAIR (Fostering Age-Inclusive Research) Trials Working Group is now advocating for the inclusion of adolescents aged >12 years in adult Phase 1 studies once the initial adult dose, toxicity and pharmacokinetics have been evaluated and inclusion of children in Phase 2–3 trials where the disease is similar in children and adults.^11^ Importantly, the US RACE for Children Act, coming into effect in August 2020, will require paediatric investigation of all drugs that are substantially relevant to the growth or progression of a paediatric cancer (see https://www.accelerate-platform.org/whyplatform/the-platform/us-pediatric-equity-act-race-children-act/). The recent experience with larotrectinib highlights the importance to pharmaceutical companies of working with HTA bodies as they develop tumour-agnostic drugs to ensure that they consider the evidence requirements that will allow timely access to patients.^1^ Marketing authorisation of more than one tumour-agnostic drug in the same class (e.g. both larotrectinib and entrectinib for TRK fusions) will in future be important to the NHS through introducing an element of competitive pricing.

In conclusion, in our view the development pathway of larotrectinib forms an important case study for the accelerated development of tumour-agnostic drugs relevant to children. Screening for relevant targets within paediatric genomic screening programmes and ongoing truly collaborative efforts between HTA and regulatory authorities, pharmaceutical companies, academic/clinical investigators and patient advocates will be crucial to help secure early access for children to such drugs in relevant clinical trials and subsequently into future standard of care.

## Data Availability

Not applicable.
